# Self-Rated Attentiveness Interacts with Transcranial Direct Current Stimulation and Noise Stimulation in Reaction Time in a Go/No-Go Task

**DOI:** 10.1155/2016/5302538

**Published:** 2016-01-14

**Authors:** Sverker Sikström, Anna-Maria Jürgensen, Maryam Haghighi, Daniel Månsson, David Smidelik, Thomas Habekost

**Affiliations:** ^1^Department of Psychology, Lund University, Paradisgatan 5, 22222 Lund, Sweden; ^2^Department of Psychology, Øster Farimagsgade 2A, 1353 København K, Denmark

## Abstract

Previous research has found that stimulating inattentive people with auditory white noise induces enhancement in cognitive performance. This enhancement is believed to occur due to a statistical phenomenon called stochastic resonance, where noise increases the probability of a signal passing the firing threshold in the neural cells. Here we investigate whether people with low attentiveness benefit to a larger extent than attentive people from stimulation by auditory white noise and transcranial direct current stimulation (tDCS). The results show, for both auditory noise and tDCS stimulation, that the changes in performance relative to nonstimulation correlate with the degree of attentiveness in a Go/No-Go task, but not in a* N*-back task. These results suggest that the benefit of tDCS may interact with inattentiveness.

## 1. Introduction

Previous research has found that inattentive people's cognitive performance selectively benefits from stimulation with auditory white noise. It has been suggested that noise may improve cognitive performance through a phenomenon called stochastic resonance (SR, [1]) where noise increases the likelihood of a signal passing the firing threshold in neural cells. This threshold is particularly high in people with low levels of attention [2]. Several studies have now found an interaction between attention and auditory stimulation in various cognitive tasks (e.g., [3, 4]). The purpose of this paper is to investigate if this interaction also occurs for transcranial direct current stimulation (tDCS), where the brain is stimulated with a weak electrical current. Previous research has found that such stimulation may increase cognitive performance in general (e.g., [5–7]).

Here we investigate whether tDCS and auditory stimulation interact with self-reported levels of attentiveness. As a measure of cognitive performance we used Go/No-Go and* N*-back tasks. These two tasks measure response inhibition and working memory capacity, respectively. Both of these dimensions are essential components of attention [8, 9]. Both the* N*-back [10] and the Go/No-Go task [11–13] are commonly used to measure those two components.

## 2. The Effect of White Noise on Cognitive Performance 

Previous research has indicated that auditory white noise may improve cognitive performance in inattentive people. Söderlund et al. [3] showed that auditory white noise leads to an increase of the attention level among people with attention deficits. In this study auditory white noise was administered in a verbal task where participants had to learn short sentences. The results showed that children with ADHD performed better in the test phase when stimulated with auditory white noise during the encoding of the sentences. In another experiment children without ADHD diagnoses, selected on their teacher's report about the children's attention level, were divided into “high attention” and “low attention” groups [4]. The children's task was to encode presented sentences with verbs and nouns while being stimulated with noise. The results showed that auditory white noise stimulation improved performance in the “low attention” group, while the “high attention” group showed decreased performance compared to the nonstimulation condition [4].

## 3. The Moderate Brain Arousal (MBA) Model

The mechanisms of how different types of stimulation of the brain can enhance cognitive performance and how this interacts with a person's attentiveness are not yet fully understood. However, an attempt to explain interactions between attentiveness, stimulation, and cognitive performance was formulated in the moderate brain arousal (MBA) model [2]. This neurophysiological model accounts for the effects of random auditory noise on cognitive performance. It is based on the idea that internal noise can be induced into the central nervous system (CNS) through the perceptive system. The brain operates at the peak of its capacity when arousal level is optimal [14]. This is modulated through the dopamine system [15]. For some individuals the natural arousal level in the CNS can be lower than optimal which can cause deficits in performance. In this case noise can enhance performance through statistical resonance (SR). SR refers to a phenomenon where the processing of a relevant signal is enhanced when random noise is added in nonlinear systems [1]. At any given moment the brain is exposed to input carrying both target signal and noise. In order to function efficiently, signals need to be detected from the noisy background. At the same time processing of noise should be inhibited. The signal-to-noise ratio (SNR) is thus an important characteristic within a cognitive system because noise can distract attention from the relevant target signal [16]. In individuals with ADHD or subclinical attentional deficits, the relation between excitatory actions as a reaction to the target signal and inhibitory actions directed at noise is disrupted, which may be caused by a malfunction of the dopamine system [2]. These individuals should profit from additional noise that would enhance performance through SR.

The MBA model has already been used successfully in understanding how people with ADHD can enhance their cognitive performance through random auditory noise (e.g., [3, 4]). The model also suggests that the threshold is higher in inattentive individuals, where the stimulation helps lowering the threshold, possibly leading to a benefit in cognitive performance.

## 4. The Effect of tDCS Stimulation on Cognitive Performance

Past research has not directly compared people with high and low attentional level in respect to the effect of tDCS stimulation on cognitive performance. But even though little is known about this interaction, several studies have examined the main effect of tDCS on attentional function. Clark et al. [6] found significant improvements in object learning when the participants were stimulated with tDCS. Each participant's brain activity was initially measured during task performance using fMRI, where the right inferior frontal cortex (rIFC) and right parietal cortex (rPC) showed higher activity during the performance of the given task. Thereafter tDCS electrodes were applied to stimulate areas that were active in the fMRI investigation. The study found significantly increased performance and learning improvements [6]. A study by Nelson et al. [17] used tDCS to enhance vigilance in adult operators. A vigilance task and a signal detection parameter task were used to measure the behavioral modes of the participants. During the tDCS and sham conditions the participant's hemispheric blood flow velocity and regional blood oxygenation were measured. Overall the results of this study showed significant performance improvement in both the vigilance and signal detection task and increased blood flow in corresponding brain areas when the participants were stimulated with tDCS. During sham condition the result did not show improved performance and lower blood flow. Other studies have also provided promising results regarding the ability of tDCS to increase attentiveness and vigilance [5–7, 17–21].

## 5. Predictions and Hypothesis

Based on the MBA model we predict a positive effect of moderate auditory noise and tDCS on cognitive performance in inattentive people. Even though the MBA model predicts a similar interaction effect for both types of stimulation, it postulates different underlying mechanisms. While auditory white noise should influence performance by introducing additional neuronal noise, we argue that tDCS influences the activation threshold [2]. According to the MBA model a neuron's sensitivity and reactivity to a signal can be enhanced by a moderate constant level of activity that is unrelated to the signal. This is due to each neuron's nonlinear threshold activation function. It can also be expected that the interaction effect will be more pronounced in tasks that are less stimulating, whereas in a more interesting and thus stimulating task additional stimulation may not be helpful. In summary, we predict a positive correlation between inattentiveness and improvement in cognitive performance both when participants are stimulated either with auditory white noise or with tDCS and this effect should be stronger in tests that are less stimulating.

To test these hypotheses we set up an experiment where participants were simulated with either auditory white noise or tDCS and compared this to a baseline condition without stimulation. In each session we systematically introduced the baseline prior to the stimulation condition, to avoid the possibility that prolonged effects of tDCS stimulation could influence performance in the baseline condition. This design allowed us to isolate the effect that is relevant to our hypothesis, which is the interaction between attentiveness and improvements in performance following stimulation relative to the baseline. However, this design precludes the possibility of studying the overall effect of stimulation, as the ordering of baseline and stimulation conditions was not counterbalanced in each session. We used a Go/No-Go test to study response inhibition and a* N*-back test to measure working memory capacity, expecting that the former test would be less challenging than the latter.

## 6. Materials and Methods

### 6.1. Design

The study followed a 1 × 3 factorial within subject design, where the participants underwent two stimulation conditions; tDCS stimulation and auditory white noise stimulation, which were compared to a baseline condition without stimulation.

### 6.2. Participants

Recruitment was conducted through notes on billboards and alerts on social media using a web-based interest application form. The application form asked for participants contact information and questions related to the exclusion criteria. Exclusions were based on self-reports of severe vision or hearing deficits with no compensatory aids, pregnancy, suffering from alcohol and/or drug addiction, diagnoses with epilepsy, borderline personality disorder, heart problems, and metal or electrical implants. The final sample consisted of 20 participants between 18 and 36 years with an average age of 26.7 years. Eight of them were women. Participants were initially informed about the purpose of the study (but not about the hypothesis), followed by a short description of the techniques and possible side effects of the stimulation. They were then asked to sign a consent form. Participants were recruited and tested on an ongoing basis.

#### 6.2.1. Attentiveness Screening

The SNAP-IV questionnaire [22] was used for assessment of participants general level of attentiveness. High scores reflect low attentiveness. This questionnaire is typically used in a clinical setting for initial screening of attentional difficulties such as ADHD/ADD. The questionnaire contains 18 claims, where 9 assess hyperactivity and the other ones evaluate the attention level. This study only used the questions measuring attention. The questions were answered through a web survey.

### 6.3. Procedure

#### 6.3.1. Cognitive Testing

Two cognitive tests, Go/No-Go and* N*-back, were administered. The Go/No-Go task measures sustained attention and response inhibition in a repetitive task. Participants were presented with a green circle on the screen. They were instructed to press a specific key as soon as possible in reaction to the target stimulus which was a purely green circle. When the circle showed a pattern, they were asked to inhibit the reaction and to not press the key. 20% of all signals presented were No-Go signals and the order of trials was randomized. Each symbol was presented for two seconds or until the participant's response. The 2-back task (in this case two back) was used to measure working memory capacity. Participants saw a continuous presentation of stimuli on the screen (1.5 seconds for each stimulus) and were instructed to press a specific key every time they saw a stimulus that was identical to the one presented two steps back. Error rates and reaction times were recorded for both tests. The tests were administered via a laptop with a separate mouse attached to it. Each test had 100 stimuli and took approximately 7–10 minutes to complete. The Go/No-Go test preceded the* N*-back test in each testing session. Before testing the instructions were presented to the participants on the screen; they were informed about the course and duration of the two test procedures. Before starting the test a practice session was administered.

The cognitive testing was performed under three conditions: nonstimulation, auditory white noise, and tDCS. The participants were invited to the lab twice, with at least one day between the testing sessions. On both days, the participants started with the nonstimulation condition. On one of the days, the nonstimulation condition was followed by the noise condition and the other day was again initiated with the nonstimulation condition but followed by the tDCS condition. The participants were randomly assigned to one of the two orders.

#### 6.3.2. tDCS

Prior to the administration of tDCS, participants were informed that they could expect a tingling sensation underneath the electrodes but that this would disappear after the power was switched off. The electrode configuration was according to the international 10–20 system. The anodal electrode was placed approximately above the right inferior frontal gyrus (rIFG), which stimulates areas F4, F8, C4, and T4. The cathodal electrode was placed approximately above the inferior orbitofrontal cortex (IOFC) [23]. TDCS was administered with an intensity of 1.5 mA and was active for one minute before the cognitive testing began in the tDCS condition. This was done so the participants could get accustomed to the sensation. The current was then active for the administration of the* N*-back and Go/No-Go tests.

#### 6.3.3. Auditory Noise

Participants were informed that the volume of the auditory noise would be about 80 decibels and were instructed to keep the headset on throughout the testing session. Auditory white noise was applied through the headphones using the iPhone app called Smartnoise.

## 7. Results

### 7.1. Overall Performance

The mean score of the SNAP-IV was 7.3 (SD = 6.08) ranging from 0 to 25, where the maximal possible score was 27. The Go/No-Go had a mean reaction time of 448 ms. However, a number of correct responses showed a ceiling effect (mean values were 98 percentage correct) and were not further analysed. The* N*-back test had a mean percentage accuracy of 80.7 and mean reaction time of 700 ms.

### 7.2. Interaction between Attentiveness and Stimulation

To test our main hypotheses regarding the interaction between attentiveness and stimulation we first subtracted performance (accuracy and reaction times) in the nonstimulation condition from the stimulation conditions. We then correlated the resulting values with the SNAP scores. Analyses were, unless otherwise specified, conducted with a significance level of 5%. For the results for accuracy and reaction times in the different conditions, please refer to Table 1.

**Figure 1:**
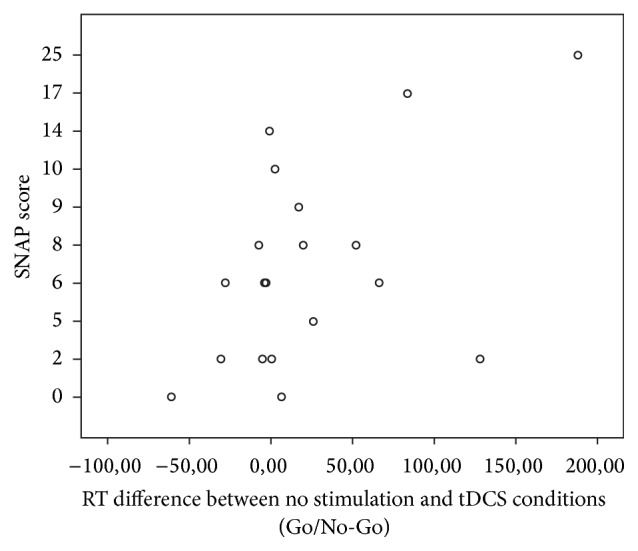
Correlation between SNAP scores and the differences between baseline and tDCS for reaction times in the Go/No-Go task.

**Figure 2:**
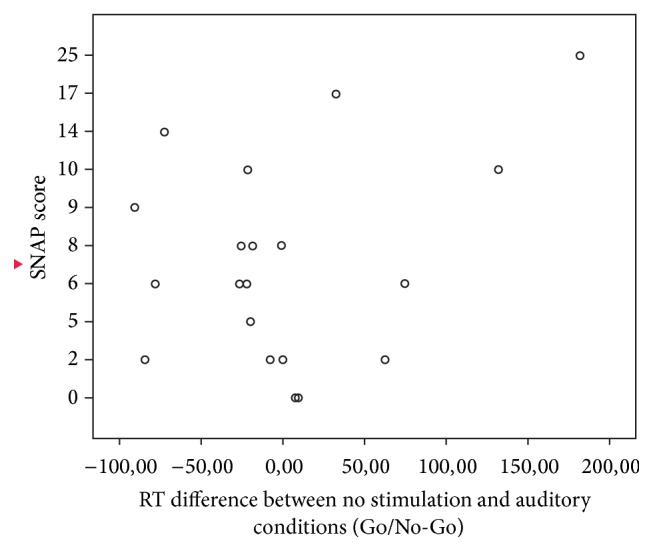
Correlation between SNAP scores and the differences between baseline and auditory stimulation for reaction times in the Go/No-Go task.

Correlation analyses were used to test for specific interactions between attentiveness (SNAP-IV score) and the changes in accuracy and reaction time between different stimulation conditions for both tests. The accuracy for the Go/No-Go task could not be interpreted due to ceiling effects. The analyses yielded two significant bivariate Pearson correlations. The difference between average reaction times in the tDCS and the nonstimulation condition correlated significantly with SNAP-IV score *r* = 0.607, *P* < 0.01 for the Go/No-Go task (Figure 1). The difference between reaction times for the Go/No-Go task in the baseline and auditory noise condition correlated significantly with the SNAP-IV score *r* = 0.414, *P* < 0.05 (Figure 2). No interaction effects with attentiveness were found between the percentage accuracy and the reaction times in the* N*-back test.

## 8. Discussion

The current study investigated the interaction between high and low attentiveness and stimulation by either tDCS or auditory noise. The study was motivated by the MBA model suggesting that both auditory noise and current stimulation interact with attentiveness on cognitive performance. According to the model, the two types of stimulation target different mechanisms. Random auditory noise adds to the internal noise in the brain which modulates the signal-to-noise ratio in favor of the signal by means of statistical resonance. Current stimulation on the other hand lowers the activation threshold of neural cells and thereby enhances detection of target signals [2]. Thus, following the suggestions of the MBA model, we expected that both tDCS and auditory white noise stimulation would interact with attentiveness. To examine our hypothesis we used two cognitive tests, namely, Go/No-Go and* N*-back. These tests were chosen to examine participants' inhibition and working memory capacity as central components of attention. Several studies (e.g., [5–7, 17–21]) have shown effects of tDCS on cognitive performance. But we are not aware of any studies where tDCS's effects were examined on inattentive versus attentive individuals in a nonclinical sample.

Our results show the expected interaction effect in the Go/No-Go test measuring inhibition, but not in the* N*-back test that was used to measure working memory capacity. The results suggest that participants who reported themselves as inattentive profited more than attentive participants from stimulation with white noise or tDCS. This interaction with attention was observed for the Go/No-Go task, but not for the* N*-back task with either white noise or tDCS stimulation.

A possible explanation for why the interaction effect was found in the Go/No-Go task, but not in the* N*-back task, could be differences between the two cognitive tasks. Based on both percentage correct levels and the general complexity of the task, it is plausible that the* N*-back task was more attentively demanding, which would lead to a higher level of arousal in the participant than for the Go/No-Go task. Thus in the* N*-back task, even participants with low general attentiveness might have performed at their individual maximum without stimulation. This could explain why no performance improvement for inattentive individuals was observed when stimulation was introduced.

## 9. Conclusion

Overall the results confirm our hypothesis derived from the MBA model. The expected interaction between low general levels of attentiveness and the benefit of external stimulation can be seen for both types of stimulation. According to the model both auditory white noise and tDCS should indeed produce a similar effect on performance. Also, the benefit of stimulation should be largest in tasks that are not in themselves cognitively arousing for the participant. This prediction was also confirmed by the results, as we found a significant interaction effect only for the task with low attentional demands, the Go/No-Go task. In that sense our data can be regarded as a first step to the verification of the MBA model for tDCS stimulation. However, further research is needed to examine this interaction.

## Figures and Tables

**Table 1:** Mean values for performance.

	Stimulation		
	Nonstimulation	Auditory noise	tDCS
Go/No-Go accuracy	97.80 (2.50)	98.25 (2.34)	98.15 (2.03)
Go/No-Go RT	456.11 (77.54)	454.79 (67.66)	433.35 (87.96)
*N*-back accuracy	75.38 (12.38)	82.10 (11.92)	84.60 (9.28)
*N*-back RT	768.15 (169.29)	677.39 (139.30)	654.24 (116.38)

*Notes. *Mean values and standard deviations in brackets; reaction times (RT) in milliseconds.
